# Does adjusting for biological maturity when calculating child weight status improve the accuracy of predicting future health risk?

**DOI:** 10.1186/s12889-021-12037-4

**Published:** 2021-11-02

**Authors:** Fiona B. Gillison, Elisabeth B. Grey, Sean P. Cumming, Lauren B. Sherar

**Affiliations:** 1 Department for Health, University of Bath, Claverton Down, Bath, BA2 7AY UK; 2 School of Sport, Exercise and Health Sciences, Loughborough University, Loughborough, UK

**Keywords:** ALSPAC, Maturity timing, Childhood obesity

## Abstract

**Background:**

The aim of this study was to assess whether adjusting the weight categorisation of children for their biological maturity status could improve the accuracy of predicting weight status and cardiometabolic risk at age 17.

**Methods:**

Data from 1525 participants (787 female) from the ALSPAC study were analysed. Participants’ weight status at age 11 was estimated using first standard chronological age and sex adjusted BMI cut-offs, and again using maturity adjusted BMI cut-offs. Each BMI category at age 11 was regressed against cardiometabolic risk score and BMI category at age 17, controlling for sex, ethnicity and socio-economic status.

**Results:**

At age 11 years, 22% of boys and 46% of girls who were categorised as overweight or having obesity based on chronological age were re-categorised into a lower BMI category after adjusting for biological maturity. Biologically adjusted BMI categories better predicted BMI category at age 17 compared with non-adjusted BMI categories (∆BIC = − 21.69); the odds of having obesity at age 17 were 18.28 times greater with each increase in BMI category at age 11. Adjusted and non-adjusted BMI status at 11 years showed equivalent accuracy in predicting cardiometabolic risk at age 17; the odds ratio of high cardiometabolic risk was 1.85, with heightened risk in boys, particularly early maturers.

**Conclusion:**

The traditional method of categorising adolescents into a BMI category may over-predict overweight and obesity, particularly in girls. Adjusting for biological maturity when estimating weight status through calculating adolescents’ BMI classification was equivalent to standard approaches in predicting other cardiovascular risk at age 17.

**Supplementary Information:**

The online version contains supplementary material available at 10.1186/s12889-021-12037-4.

## Introduction

Increasing rates of childhood obesity are a significant concern for public health [1, 2] due to the association of obesity with poor health outcomes that can track into adulthood [3]. National surveillance of childhood overweight and obesity can be a central part of obesity strategies, for example the UK [1, 2], Netherlands [4], Germany, Australia and New Zealand [5] all measure children in early adolescence (around age 11). However, the use of BMI in diagnosing overweight and obesity in children has been criticised as its sensitivity varies considerably, especially around the time of the adolescent growth spurt where sex specific differences in body fat accumulation occur [6, 7]. Obtaining a measure of excess weight in childhood that accurately predicts future health risk is challenging [8].

Recent research has explored how BMI cut-offs can be adjusted to account for ethnic minority groups to increase reliability of population-level estimates of obesity [9, 10]. There is also a rationale for adjusting to account for the different ages at which children enter puberty; this can vary by as much as 6 years. The variability in the timing of growth (i.e. the age at which a maturity event, such as a growth spurt, occurs) and tempo of growth (i.e. the rate at which each maturity event is attained) can result in large differences in lean and fat mass between youth of the same chronological age [11]. For example, girls who enter puberty in advance of their peers will naturally be expected to possess greater absolute and relative fat mass, as a result, they may also present higher BMI values. Consequently, the differential timing of biological maturity between children of the same chronological age may contribute to the varied sensitivity of BMI for diagnosing future obesity, overweight and cardiometabolic risk [3].

Adjustment for the timing of biological maturity can be made using validated prediction methods that only require the height of parents and children, along with the child’s age and sex, most of which is available through existing cohort study databases (i.e., the Khamis-Roche method [12]). Recent research in 407 UK children aged 9–11 suggests that if weight categorisation was adjusted for a child’s maturity status, up to 32% of girls and 15% of boys initially classified as overweight would be reclassified as healthy weight [13]. A further 11 and 8% of girls and boys initially classified as having obesity would be classified as overweight if maturity was taken into account. This finding is of particular importance as it suggests that the prevalence of overweight and obesity in British youth may be overestimated. What is not clear, however, is the extent to which adjusting weight classification for differences in biological maturation impacts the association between adolescent BMI and future health status. It is possible that those individuals who could be reclassified from overweight/obese to normal weight during childhood when biological maturity timing is taken into account are still at greater risk for negative health outcomes as adults. This is an important relationship to establish before recommendations about adapting weight classification for adolescents can be made.

Researchers have explored the relationship between obesity and timing of puberty, to ascertain whether the association observed between the two is causal. Overall the research findings in this area are heterogenous [14, 15]; a 2017 systematic review including 11 cohorts studies including 4841 participants found only weak evidence for a causal link in girls, and no evidence of a link in boys, in part limited by the lack of good quality studies with standardised measure of both pubertal status and obesity [14]. A more recent longitudinal study of over 14,000 US youth did, however, find earlier maturation to be associated with higher BMI values in young adulthood for both males and females [16]. While we acknowledge the potential link between the two factors, given the uncertainty about the strength and size of effect, and the independent aims of the present study, we do not account for it in our analyses. This study aims to investigate whether adjusting for biological age in obesity categorisation in early adolescence more accurately predicts weight status (as estimated through BMI classification) and cardiometabolic risk at age 17.

## Methods

### Design and participants

Data came from the Avon Longitudinal Study of Parents and Children (ALSPAC [17, 18]). For this population-based birth cohort study, pregnant women resident in Avon, UK with expected dates of delivery 1st April 1991 to 31st December 1992 were invited to take part in the study. Of the initial 14,541 pregnancies enrolled,^1^ there were a total of 14,676 foetuses resulting in 14,062 live births and 13,988 children who were alive at 1 year of age. The present study analysed data from 1525 children for whom height and weight measurements were recorded during clinical assessments at age 11 and 17; representing 12.4% of the total sample eligible for follow-up at age 17. Children who completed follow-up assessments were more likely to be white, female and less likely to be eligible for free school meals than the eligible sample, and more likely to have higher educational attainment than the national average. Participants or their guardians provided written informed consent, and ethical approval for the study was obtained from the ALSPAC Ethics and Law Committee and the Psychology Research Ethics Approval Committee at the University of Bath (ref: PREC 18–166). The research adheres to the tenets of the Declaration of Helsinki.

### Measures

Full detail of all measures is provided in Boyd et al. [17]. Please note that the study website (http://www.bristol.ac.uk/alspac/researchers/our-data/) contains details of all the data that is available through a fully searchable data dictionary and variable search tool.

#### Height and weight

Data were collected on two occasions in clinical assessments following standardised procedures when the children were approximately 11 years and 17 years old. Height was measured to the last complete mm using a Harpenden stadiometer and a Tanita Body Fat Analyser was used to measure weight to the nearest 50 g. Children wore light clothing and no shoes for the assessments.

#### Cardiometabolic risk

In the clinical assessment at 17 years, fasting blood samples were taken from which levels of plasma glucose, high-density lipoprotein (HDL) and low-density lipoprotein (LDL) were measured (all mmol/L). Systolic and diastolic blood pressure (SBP and DBP, respectively) were measured twice in succession while participants were at rest in a seated position, with arms supported, using an appropriately sized cuff and DINAMAP 9301 machine; the mean of the two values was then calculated. Mean arterial pressure (MAP) was estimated using the formula: (SBP + 2DBP)/3.

Composite indicators have been validated for use with children [19] to provide a more comprehensive indicator of health risk than any single indicator alone, enabling us to consider the clustering of risk factors while being statistically robust (i.e., avoiding repeated analyses for individual indicators and allowing us to retain the data as continuous which is more sensitive than a dichotomous approach. Therefore, in the present study a continuous cardiometabolic risk score (CMR) was created from the z-scores of these variables, first converting HDL scores to their reciprocals, according to the following equation:

$$ \mathrm{CMR}=\left(1/{\mathrm{HDL}}_{\mathrm{Z}-\mathrm{score}}\right)+{\mathrm{LDL}}_{\mathrm{Z}-\mathrm{score}}+{\mathrm{fasting}\ \mathrm{glucose}}_{\mathrm{Z}-\mathrm{score}}+{\mathrm{MAP}}_{\mathrm{Z}-\mathrm{score}} $$

#### Covariates

Demographic covariates were recorded after enrolment in the ALSPAC study during the early years of the child’s life through questionnaires completed by mothers, and included child sex, ethnicity (collapsed into white vs. non-white due to low numbers of non-white participants) and mother’s socioeconomic group based on occupation prior to the child’s birth (categorised into four groups: employers, managers and professionals; intermediate non-manual and skilled/supervisor manual; junior non-manual; and unskilled manual). Missing data precluded the inclusion of father’s occupation.

### Classification of biological maturity timing and BMI category

#### Biological maturity timing

Maturity timing was classified through calculating the difference between a child’s chronological age, and their estimated biological age. Children whose biological age was 1 year or more greater than their chronological age were classified as early maturers, and children whose biological age was 1 year or more lower than their chronological age were classified as later maturers. A discrepancy of + − 1 year between chronological and biological age has routinely been used to classify adolescents as early or late maturing and closely corresponds to the criterion of + − 1 standard deviation from the population norms [20].

Biological age was estimated using the Khamis-Roche method to estimate the percentage of adult height attained, based on a child’s height at the point of measurement relative to mid-parent height [12]. The higher the percentage of adult height attained, the more biologically mature the individual [21]. Each child’s percentage of adult height attained was compared against age- and sex-specific reference standards generated in the UK using the 1990 growth reference data [22]. Age- and sex-specific percentage values were calculated on the basis of mean values for stature attained at each age interval, and the mean values for stature attained at and above 18 years of age (177.6 cm in males; 163.7 cm in females). The Khamis Roche was selected as it is non-invasive, acceptable to children and parents, and has been shown to serve as a valid and reliable estimate of growth and maturation in North American and European youth [23, 24].

#### BMI category

BMI (weight in kg/height in meters^2^) was calculated at each time point (11 and 17 years) and transformed into a BMI percentile relative to the 1990 UK reference values matched to an individual’s age (in months) and sex [22]. To align with population level cut-off scores chronological weight status was assigned by categorising children with a BMI of ≥2nd percentile and < 85th percentile as a healthy weight, ≥85th percentile and < 95th percentile as overweight, and ≥ 95th percentile as having obesity. Underweight children are reported in the sample description but were excluded from further analysis as these were low in number and outside the central research question.

A second, biological maturity adjusted BMI percentile was calculated by comparing BMI to gender-specific biological age-matched norms, and used to categorise each individual as underweight, normal weight, overweight or having obesity according to percentiles set out above. To do this we matched individuals to the reference group within the 1990 dataset that corresponded to their percentage of adult height achieved, rather than their chronological age, and calculated their BMI percentile accordingly. For example, if girls on average reach 91·5% of predicted adult stature at age 12, a girl of 10·5 years of age reaching this % would be considered an early maturer. Accordingly, her adjusted BMI category would be achieved through calculating her BMI percentile relative to girls with the same biological maturational age of 12·0 years, rather than her chronological age of 10.5 years.

### Analyses

Statistical analyses were conducted using SPSS version 26 [25]. Separate multinomial logistic regression models were used to examine whether weight status, estimated through BMI category at age 11 (unadjusted and BMI adjusted), predicts (a) BMI category and (b) cardiometabolic risk (low; high, categorised via a median split) at age 17. The covariates were sex, ethnicity and maternal socio-economic status. Deviance dispersion scaling was applied to the logistic regression models to overcome potential overdispersion from repeated measures.

The fit of each model was judged using the Akaike information criterion (AIC) and the Bayes information criterion (BIC), with lower scores indicating better fitting models [26].

## Results

The sample comprised 1525 participants (738 boys, 787 girls), 98% were white and 49% were from households in which mothers reported manual or non-professional job grades. Table 1 presents the descriptive weight and biological maturity data for the sample at age 11 years. The differences in the number of children in each cell of Table 1 using standard (chronological) versus adjusted (biological) cut points represent the movement down a category for 31% of children (22% of boys, and 46% of girls) who were categorised as overweight or having obesity. Table 2 displays the weight and health indicator data at age 17 years.

**Table 1:** Sample description at age 11

	Boys Mean (SD)	Girls Mean (SD)	Full sample Mean (SD)
Age (years)	11·75 (0·21)	11·75 (0·20)	11·75 (0·20)
BMI (kg/m^2^)	18·45 (3·00)	18·65 (3·14)	18·55 (3·07)
BMI category using standard (chronological) age-matched norms			
	N (%)	N (%)	N (%)
Underweight	16 (2)	15 (2)	31 (2)
Healthy weight	522 (71)	619 (79)	1141 (75)
Overweight	99 (13)	71 (9)	170 (11)
With obesity	101 (14)	82 (10)	183 (12)
BMI category using maturity adjusted (biological) age-matched norms			
Underweight	13 (2)	17 (2)	30 (2)
Healthy weight	544 (74)	650 (83)	1194 (78)
Overweight	99 (13)	78 (10)	177 (12)
With obesity	82 (11)	42 (5)	124 (8)
Biological maturity indicators			
% adult height achieved (SD)	84% (1·91)	93% (3·65)	88% (5·37)
Early maturers	59 (8)	252 (32)	311 (20)
Average maturers	665 (90)	493 (63)	1158 (76)
Late maturers	14 (2)	42 (5)	56 (4)

Although children who were underweight at age 11 were excluded from the primary analyses, they are presented here to demonstrate potential movement between classifications with adjustment

**Table 2:** Health risk information at age 17

	Boys Mean (SD)	Girls Mean (SD)	Total Mean (SD)
Age (years)	17·70 (0.35)	17·72 (0.37)	17·71 (0.36)
Cardiometabolic risk score^a^	0·62 (2.25)	-0·61 (2.18)	0·01 (2.30)
BMI category	N(%)^b^	N(%)^b^	N(%)^b^
Healthy weight	471 (75)	609 (80)	1080 (78)
Overweight	81 (13)	84 (11)	165 (12)
With obesity	80 (13)	68 (9)	148 (11)

^a^Lower values indicate lower risk

^b^reported % are of participants providing data for each variable, not the full sample

### Biological maturity timing and BMI status in late adolescents

Using non-adjusted cut-offs, early maturing children were more than three times as likely to be classified as overweight or having obesity than average or late maturing children (OR overweight or obesity = 3·71; OR obesity = 5·04) (Supplementary Table 1). These odds decreased slightly at age 17, but early maturers were still over twice as likely to either be overweight or have obesity than average or late maturers, (OR = 2·63), and over three times more likely to be categorised as having obesity (OR = 3·58). Using adjusted cut-offs, the odds of being overweight or having obesity as an early maturer were slightly lower at 2·79.

Sixty-two percent of boys and 55% of girls with obesity at age 11 remained obese at age 17 using non-adjusted cut-offs, but this rose to 69–70% for both sexes when biological maturity adjusted categories were used (Table 3). The distribution was largely similar for boys and girls.

**Table 3:** Distribution of those classified as overweight or obese at age 11 by chronological or biological age adjusted BMI categories at age 17

		Age 17	
Age 11	Healthy weight	Overweight	Obese
BMI status using standard (chronological) age-matched norms			
Healthy weight	**963 (90%)**	85 (8%)	17 (2%)
% boys/girls	91/90	8/8	1/2
Overweight	79 (49%)	**50 (31%)**	33 (20%)
% boys/girls	46/52	32/30	22/18
With obesity	38 (23%)	30 (18%)	**98 (59%)**
% boys/girls	19/28	19/17	62/55
BMI status using maturity adjusted (biological) age-matched norms			
Healthy weight	**990 (89%)**	101 (9%)	26 (2%)
% boys/girls	90/88	9/9	2/3
Overweight	76 (45%)	**46 (27%)**	46 (27%)
% boys/girls	46/44	29/26	25/30
With obesity	15 (14%)	18 (17%)	**76 (70%)**
% boys/girls	11/19	19/11	69/70

Figures in bold indicate the expected classification of the majority of children, if BMI status proved stable over time. For full-sample chronological cut-offs χ^2^ = 634 (4, 1) *p* < 0·001, for biological cut-offs χ^2^ = 611 (4, 1) *p* < 0·001

### Prediction of health outcomes at age 17

The fit statistics for models predicting health outcomes at age 17 based on adjusted and non-adjusted categories at age 11 are set out in Supplementary Table 2.

#### Weight status

The model using biologically adjusted BMI categories showed meaningfully better fit than that with chronological BMI categories (∆BIC = −21·69), and correctly classified 82% of cases. With each increase in BMI category at age 11 (i.e., from healthy weight to overweight to obese), the odds of being overweight at age 17 were 4·19 times greater, and the odds of having obesity were 18·28 times greater.

#### Cardiometabolic risk

There was little difference between the fit of the two models (∆BIC < 2) in predicting cardiometabolic risk, both correctly classified 63% of cases. Boys were twice as likely as girls to have elevated cardiometabolic risk at age 17. With each increase in unadjusted BMI category at age 11, the odds of having higher cardiometabolic risk at age 17 rose by 1·85.

### Interactions between biological maturity timing and BMI status

The potential interaction effects of (i) maturity timing (early, on-time or late) by BMI status, and (ii) maturity timing by sex were added to the models, while ethnicity and socio-economic status were removed for parsimony.

Both of the interaction terms were significant predictors of obesity at age 17, whether using the unadjusted (Chi^2^ (12)=520·29, *p* < 0·001) or the biologically adjusted BMI categories (Chi^2^ (12) = 494·64, *p* < 0·001) at age 11. The adjusted model showed meaningfully better fit (∆BIC = 2·72); of children who were overweight or had obesity at age 11 (using adjusted cut-offs), early and on-time maturers had a higher BMI than their late maturing peers by age 17 (Supplementary Fig. 1). There was a trend towards early maturing boys being more at risk of higher BMI at age 17 than early maturing girls (Supplementary Fig. 2).

Both the interaction terms were also significant predictors of cardiometabolic risk at age 17, whether using the unadjusted (Chi^2^ (6) = 80·16, *p* < 0·001) or the biologically adjusted BMI categories (Chi^2^ (6) = 88·65, *p* < 0.001) at age 11. For cardiometabolic outcomes, the model using unadjusted BMI categories showed meaningfully better fit (∆BIC = 4·76). Cardiometabolic risk at age 17 was higher for all children who were overweight or had obesity at age 11 than for healthy weight children, regardless of biological maturity timing. The interaction effect suggested that early maturity conferred lower risk only in children with a healthy weight at age 11 (Supplementary Fig. 3). Regardless of BMI category at age 11, there was a greater, negative effect of early maturation (compared with average maturation) on cardiometabolic risk at age 17 in boys than girls (Supplementary Fig. 4).

## Discussion

In this sample of 1525 participants, adjusting for biological maturity by using a child’s biological age to determine their BMI percentile resulted in the reclassification of 31% of children initially classified as overweight or having obesity, into a lower (healthier) weight category. This is consistent with previous findings with a different UK sample [13]. Biological-age adjusted BMI categories proved significantly better predictors of later BMI status (at age 17) than standard chronological-age matched categories, although the two approaches were equivalent in the degree to which they predicted cardiometabolic risk. While the finding that early maturing children are on average at greater risk of current [27] and lifetime [28] obesity than those maturing late or on time has been documented, the relationship between early obesity, biological maturity timing and adulthood health risk is less certain. This study therefore provides additional longitudinal evidence of these effects. Our finding that boys were at double the risk of later cardiometabolic risk compared with girls contrasts with previous work [29]. This difference could be due to variation in how early maturity is measured and defined, and in the age at which weight and cardiometabolic outcomes are assessed.

National surveillance of childhood overweight and obesity is a central part of several nations’ obesity strategies [2, 4, 5]. Our findings suggest that using biological age cut-offs for weight categorisation of children as an adjunct to age-based standards could help to more accurately predict overweight and obesity in adolescence, and thus have the potential for large impact through improving the accuracy of such public health surveillance and screening programmes. Using biological cut-offs could also be beneficial by allowing public health teams to better identify those children at greatest risk of later obesity and poor cardiometabolic health (i.e., early maturing children, particularly males).

Reducing the number of misclassifications is also important in reducing potential unintended negative consequences of weight classification on children’s wellbeing. Previous work highlights that early focus on a child’s weight by parent or child is associated with potentially detrimental parenting behaviours (e.g. restrictive feeding practices) and poorer child self-concept [30, 31]. Parents often report concern for negative effects on their child’s wellbeing on receipt of unexpected feedback that a child is overweight from national measurement schemes [32–34]. Improving measurement accuracy to reduce the number of children who are incorrectly classified is, therefore, important both to better identify children who might most benefit from additional support, and in minimising the risk of potential stigmatisation and/or unintended consequences.

It is also important to consider potential risks of taking this alternative approach, that is, whether adopting this adjusted classification approach would reduce adolescents’ access to effective treatment. Parental recognition of overweight and obesity in their children is a prerequisite for both independently making changes to diet and physical activity at home and seeking treatment. Most families receiving a referral to children’s weight management services do not accept the offer, which is commonly attributed to denial or lack of recognition that a child is overweight [35]. Parental recognition of a child’s weight status is poor even following confirmation of measurement from school nurse-led programmes [36], as some parents do not believe these weight classifications, often citing the failure to account for biological maturity within the measurement process as a reason for not trusting them [33, 34, 37–39]. This suggests that the costs in lost opportunity for support among adolescents reclassified by the present approach is very low. Conversely, if awareness and acceptance among parents of children who remain in the overweight categories following adjustment is increased by receiving more tailored feedback, this would be a benefit; particularly given that the children who are not reclassified following adjustment are by definition those further above the borderlines of each category.

A key strength of this study was the use of a large cohort of children, with data collected objectively, and the inclusion of a comprehensive cardiometabolic health profile. Using data collected when the children were age 11 was important as this is the age at which children in England are measured for the NCMP and, as puberty starts to affect many children around this age, parents can lack trust in the weight assessments [33, 37, 38]. However, the sample was limited in its ethnic diversity; standard chronological age cut-offs for BMI are known to be inaccurate for body fatness (and thus obesity status) estimation in children of South Asian and Black ethnicity [9, 10]. Further work is therefore needed to investigate whether biological age cut-offs are more accurate and lead to better prediction of future health among children of various ethnicities. While the Khamis-Roche method is a simple and non-invasive method for predicting biological maturity which could be integrated in national programmes relatively easily (e.g. [40]), it has not been validated within UK samples [13] and will contain more error than objective measures (such as attained age at peak height velocity). Furthermore, the Khamis-Roche approach requires the collection of data from children’s biological parents which is not possible for all children. A limitation with the continuous cardiometabolic risk score used in this paper is that it is sample-specific (i.e., it does not align to clinical at risk cut-points); however, similar cardiometabolic cluster scores calculated during early adolescence have previously been shown to have good sensitivity and specificity with metabolic syndrome during adolescence [19] and predict later (early 20s) cardiovascular risk [41]. A further limitation was that we did not control for cardio-metabolic risk scores at age 11.

There is a need for future studies to use serial data spanning late childhood through adolescence to understand the impact of growth and development (e.g. the timings of the adolescent growth spurt in height and weight) on BMI classifications. Using traditional (i.e. chronological age and gender specific) cut-offs for children’s weight has shown poor predictive ability of adult morbidity [3] and so alternative methods may be needed. This study investigated the prediction of cardiometabolic health at age 17 from childhood weight status at age 11; future studies could examine whether using biological age cut-offs and considering maturity timing is helpful in prediction of broader health status at early, middle and late adulthood, which will be possible as birth cohorts (such as ALSPAC) continue to age. In the present study, biological cut-offs were equivalent to standard chronological age cut-offs in predicting cardiometabolic risk at age 17. Future research should investigate whether biological maturity adjusted BMI categories either at age 11 or 17 can predict later adult morbidities. For reasons of parsimony and availability of data, only a limited set of moderating variables were included in our models to predict obesity and cardiometabolic health. Future work should consider factors such as family history of obesity and cardiometabolic disorders, which are known to predispose individuals to these conditions [42, 43].

### Implications for practice

The approach reported here of adjusting weight classification on the basis of biological maturity timing would be simple to incorporate into practice, through the use of a conversion calculator (i.e., a spreadsheet linking a user interface to normative datasets through an embedded formula). The calculator could be available on or offline, to be used in situ (e.g., during a face to face appointment) or applied to a database post measurement. Similarly, it would be feasible to add this function to current BMI calculators available online for school nurse teams and parents to use when considering feedback from the National Child Measurement Programme (https://www.nhs.uk/live-well/healthy-weight/bmi-calculator/).

## Conclusion

In summary, the results of the present study provide some impetus for considering the effects of variation in biological maturity when assessing the weight status of an adolescent and will hopefully fuel the debate as to whether classifications based on age and sex alone are the most appropriate in national obesity screening programmes.

## Supplementary Information

**Additional file 1: Figure 1.** BMI at age 17 against weight status at age 11, according to maturity timing. **Figure 2.** BMI at age 17 against sex, according to maturity timing. **Figure 3.** Cardiometabolic risk at age 17 against weight status at age 11, according to maturity timing. **Figure 4.** Cardiometabolic risk at age 17 against sex, according to maturity timing. **Supplementary Table 1.** Distribution of children across maturity and BMI categories at age 11 and 17. **Supplementary Table 2.** Outcomes of models.

## Data Availability

The informed consent obtained from ALSPAC participants does not allow the data to be made freely available through any third party maintained public repository. However, data used for this submission can be made available on request to the ALSPAC Executive. The ALSPAC data management plan describes in detail the policy regarding data sharing, which is through a system of managed open access. Full instructions for applying for data access can be found here: http://www.bristol.ac.uk/alspac/researchers/access/. The ALSPAC study website contains details of all the data that are available: http://www.bristol.ac.uk/alspac/researchers/our-data/.

## Footnotes

1 for these at least one questionnaire has been returned or a “Children in Focus” clinic had been attended by 19/07/99
