# Supplementary material for: Does adjusting for biological maturity when calculating child weight status improve the accuracy of predicting future health risk?
Source: BMC Public Health. 2021 Nov 2;21:1979. doi: 10.1186/s12889-021-12037-4 (PMC8561871; doi:10.1186/s12889-021-12037-4)
Supplement: Supplementary file 1 — Additional file 1: Figure 1. BMI at age 17 against weight status at age 11, according to maturity timing. Figure 2. BMI at age 17 against sex, according to maturity timing. Figure 3. Cardiometabolic risk at age 17 against weight status at age 11, according to maturity timing. Figure 4. Cardiometabolic risk at age 17 against sex, according to maturity timing. Supplementary Table 1. Distribution of children across maturity and BMI categories at age 11 and 17. Supplementary Table 2. Outcomes of models. [file 12889_2021_12037_MOESM1_ESM.docx]

# Does adjusting for biological maturity when calculating child weight status improve the accuracy of predicting future health risk?

Fiona B Gillison^1*^ (PhD), Elisabeth B Grey^1^ (PhD), Sean P Cumming^1^ (PhD), Lauren B Sherar^2^ (PhD)

Supplementary figures and table

Figure 1. BMI at age 17 against weight status at age 11, according to maturity timing


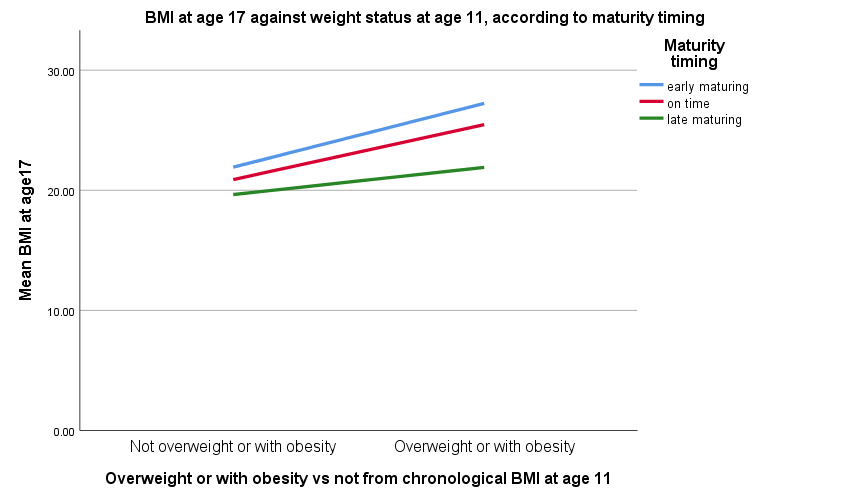


Figure 2. BMI at age 17 against sex, according to maturity timing


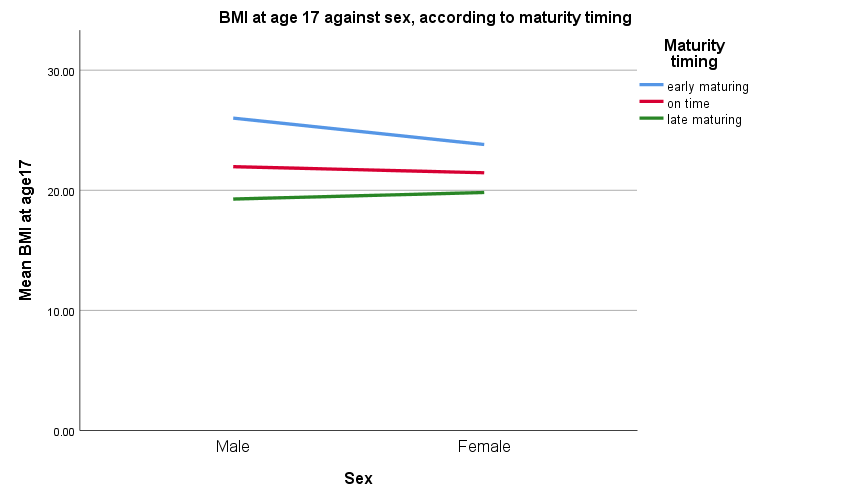


Figure 3. Cardiometabolic risk at age 17 against weight status at age 11, according to maturity timing


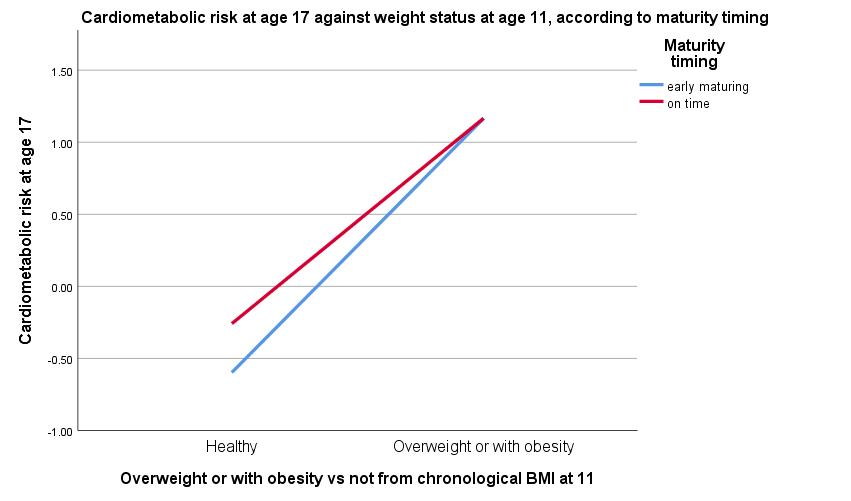


N.B. Late maturing does not appear in the graph as there were too few children (n=1) in the late mature and overweight/obesity category

Figure 4. Cardiometabolic risk at age 17 against sex, according to maturity timing


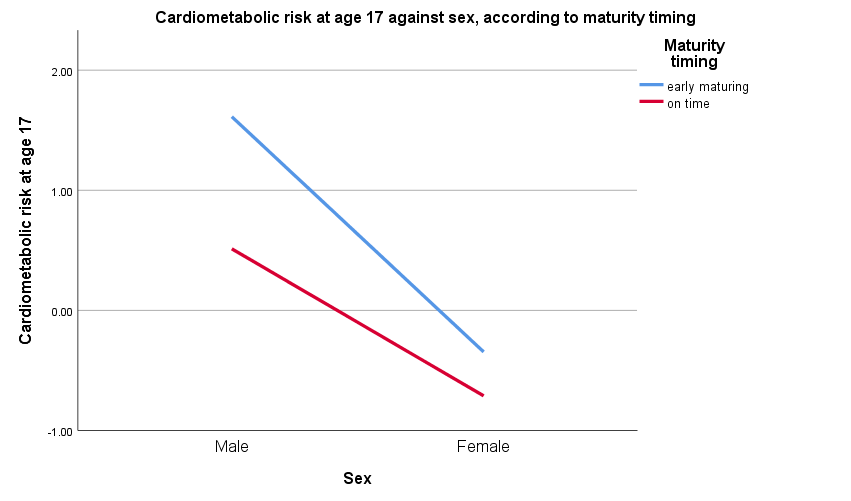


N.B. Late maturing does not appear in the graph as there were too few children (n=14) in the late mature and male category

Supplementary Table 1. Distribution of children across maturity and BMI categories at age 11 and 17

|  | Early | On time | Late | Total |
| --- | --- | --- | --- | --- |
| BMI category at 11^a^ | |  |  |  |
| healthy weight |  |  |  |  |
| chronological age | 173 (56%) | 917 (79%) | 51 (91%) | 1141 (75%) |
| biological age | 202 (65%) | 939 (81%) | 53 (95%) | 1194 (78%) |
| overweight |  |  |  |  |
| chronological age | 47 (15%) | 122 (11%) | 1 (2%) | 170 (11%) |
| biological age | 61 (20%) | 115 (10%) | 1 (2%) | 177 (12%) |
| with obesity |  |  |  |  |
| chronological age | 91 (29%) | 92 (8%) | 0 (0%) | 183 (12%) |
| biological age | 48 (15%) | 76 (7%) | 0 (0%) | 124 (8%) |
| TOTAL | 311 (20%) | 1158 (76%) | 56 (4%) | 1525 |
| BMI category at 17 ^b^ | |  |  |  |
| healthy weight | 189 (63%) | 869 (82%) | 52 (95%) | 1110 (78%) |
| overweight | 45 (15%) | 119 (11%) | 1 (2%) | 165 (12%) |
| with obesity | 66 (22%) | 80 (8%) | 2 (4%) | 148 (10%) |
| TOTAL | 300 (21%) | 1068 (75%) | 55 (4%) | 1423 |

Notes: Although children who were underweight at age 11 were excluded from the primary analyses, they are presented here to demonstrate potential movement between classifications over time; *^a^* χ^2^ = 143·25 (df 1,6) p<0·001 ; *^b^* χ^2^ = 69·78 (df 1,4) p<0·001

Supplementary table 2: Outcomes of models

|  | Model based on chronological weight categories | | | | Model based on biological weight  categories | | Model with maturity timing and interaction terms^#^ | |
| --- | --- | --- | --- | --- | --- | --- | --- | --- |
|  | Model fit  (χ^2^) | | | Significant predictors^‡^ (OR, 95% CI) | Model fit (χ^2^) | Significant predictors^‡^ (OR, 95% CI) | Model Fit  (χ^2^ ) | Significant predictors^‡^ (OR, 95% CI) |
| *Physical health risk indicators* | | | |  |  |  |  |  |
| **Weight at age 17** | 438.65 (12), p<0.001 | | | OR overweight vs healthy weight ↑ with increase in weight category at 11= 3.61 (2.65 to 4.91)  OR with obesity vs healthy weight ↑ with increase in weight category at 11= 14.45 (9.80 to 21.29) | 410.12 (12), p<0.001 | OR overweight vs healthy weight ↑ with increase in weight category at 11= 4.19 (3.09 to 5.70)  OR with obesity vs healthy weight ↑ with increase in weight category at 11= 18.28 (12.85 to 26.00) | 494.64 (12), p<.001 | - Overweight vs healthy weight ↑ maturity timing x sex = 3.90 (1.12 to 13.63); - maturity timing x overweight/obesity status at age 11 = 0.09 (0.05 to 0.19).   Obese vs healthy weight ↑  maturity timing x sex = 141.14 (47.06 to 423.29); maturity timing x overweight/obesity status at age 11 = 0.01 (0.003 to 0.011); |
| ∆BIC between chron. and bio. Models* | | |  | -22 | | | -2.72 | |
| % correct classified cases | | |  | 82% biological weight model | | | 82% biological weight model | |
| **CMR age 17** | 73.62 (6), p<0.001 | | | OR for ↑ CMR with increase in weight category at 11= 1.85 (1.49 to 2.29)  OR for ↑ CMR if male = 2.39 (1.82 to 3.15) | 74.86 (6), p<0.001 | OR for ↑ CMR with increase in weight category at 11= 2.05 (1.55 to 2.70)  OR for ↑ CMR if male = 2.33 (1.72 to 3.16) | 80.16 (6), p<0.001 | ↑ CMR   - maturity timing x sex = 3.24 (1.91 to 5.49); maturity timing x overweight/obesity status at age 11 = 0.33 (0.22 to 0.48). |
| ∆BIC between chron. and bio. Models* | |  | | 1.59 | | | 4.76 | |
| % correct classified cases | |  | | 63% chronological weight model (models equivalent) | | | 63% chronological weight model | |

Notes: *∆BIC is considered meaningful if >2, and large if >10 (ref), ^‡^ p<0.05, ^#^ for parsimony, only model fit and significant predictors for the best fitting models are given in this column (i.e. the model using biological age-matched norms for weight status at 17, and the model using chronological age-matched norms for CMR at 17).
